# Supplementary figures and images for: The implications of small stem cell niche sizes and the distribution of fitness effects of new mutations in aging and tumorigenesis
Source: Evol Appl. 2016 Mar 8;9(4):565–82. doi: 10.1111/eva.12361 (PMC4831459; doi:10.1111/eva.12361)

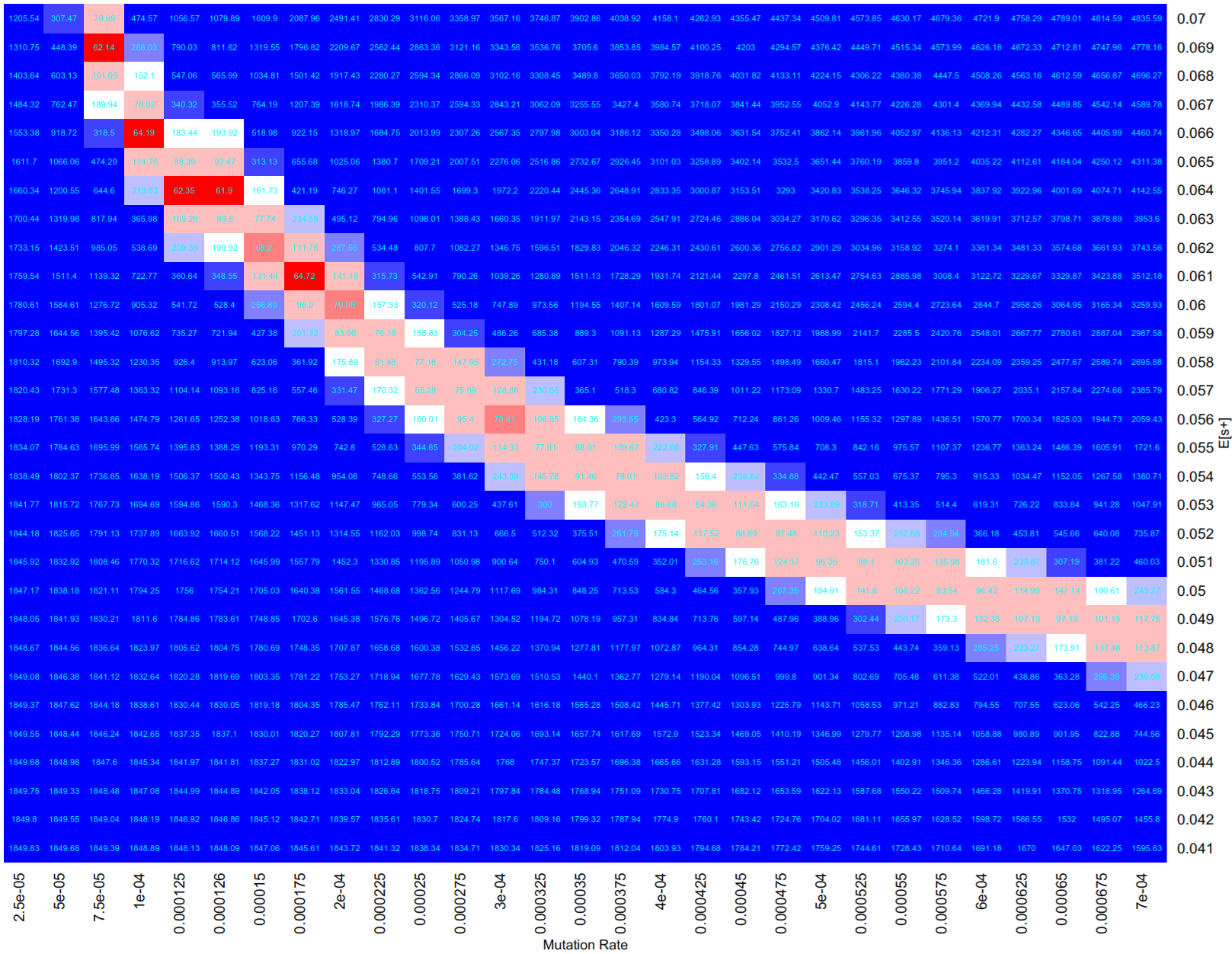

Supplement: Supplementary file 1 — Figure S1. Heat map depicting values for the least squares analysis of predicted tumor incidence and human tumor incidence data for the exponential beneficial DFE on division rate scenario. [file EVA-9-565-s001.png]

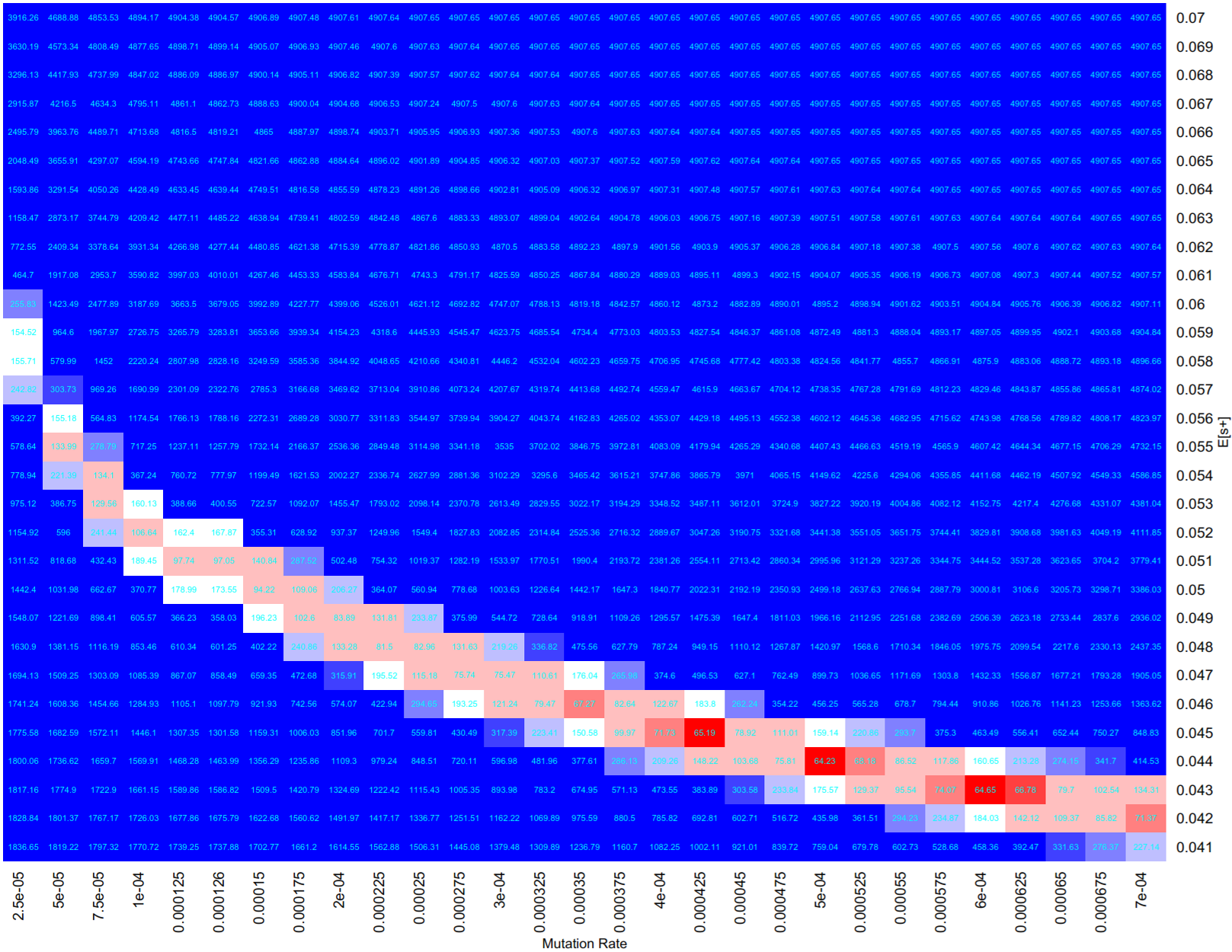

Supplement: Supplementary file 2 — Figure S2. Heat map depicting values for the least squares analysis of predicted tumor incidence and human tumor incidence data for the power‐law beneficial DFE on division rate scenario. [file EVA-9-565-s002.png]

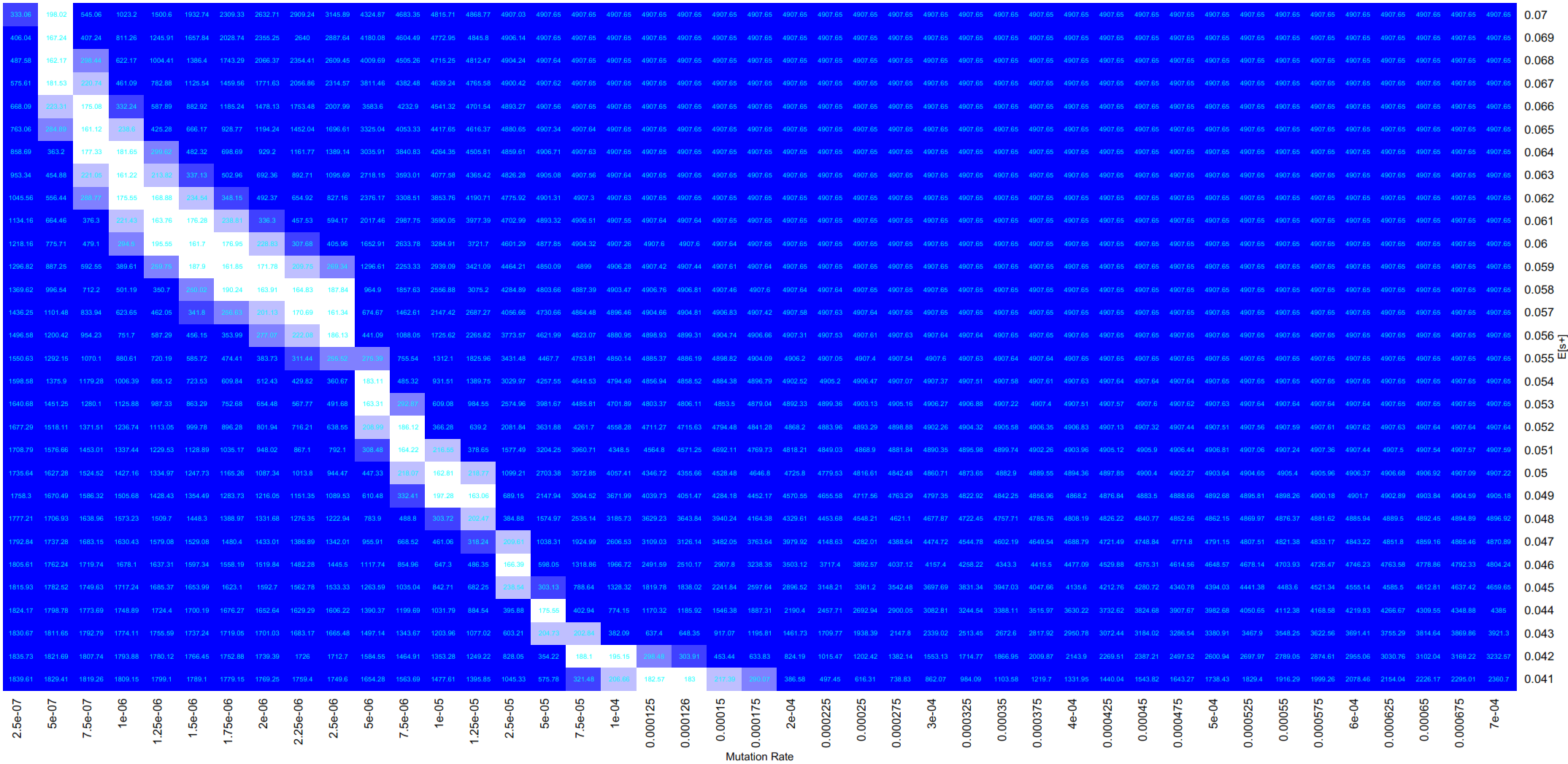

Supplement: Supplementary file 3 — Figure S3. Heat map depicting values for the least squares analysis of predicted tumor incidence and human tumor incidence data for the mutations affecting differentiation rate scenario. [file EVA-9-565-s003.png]

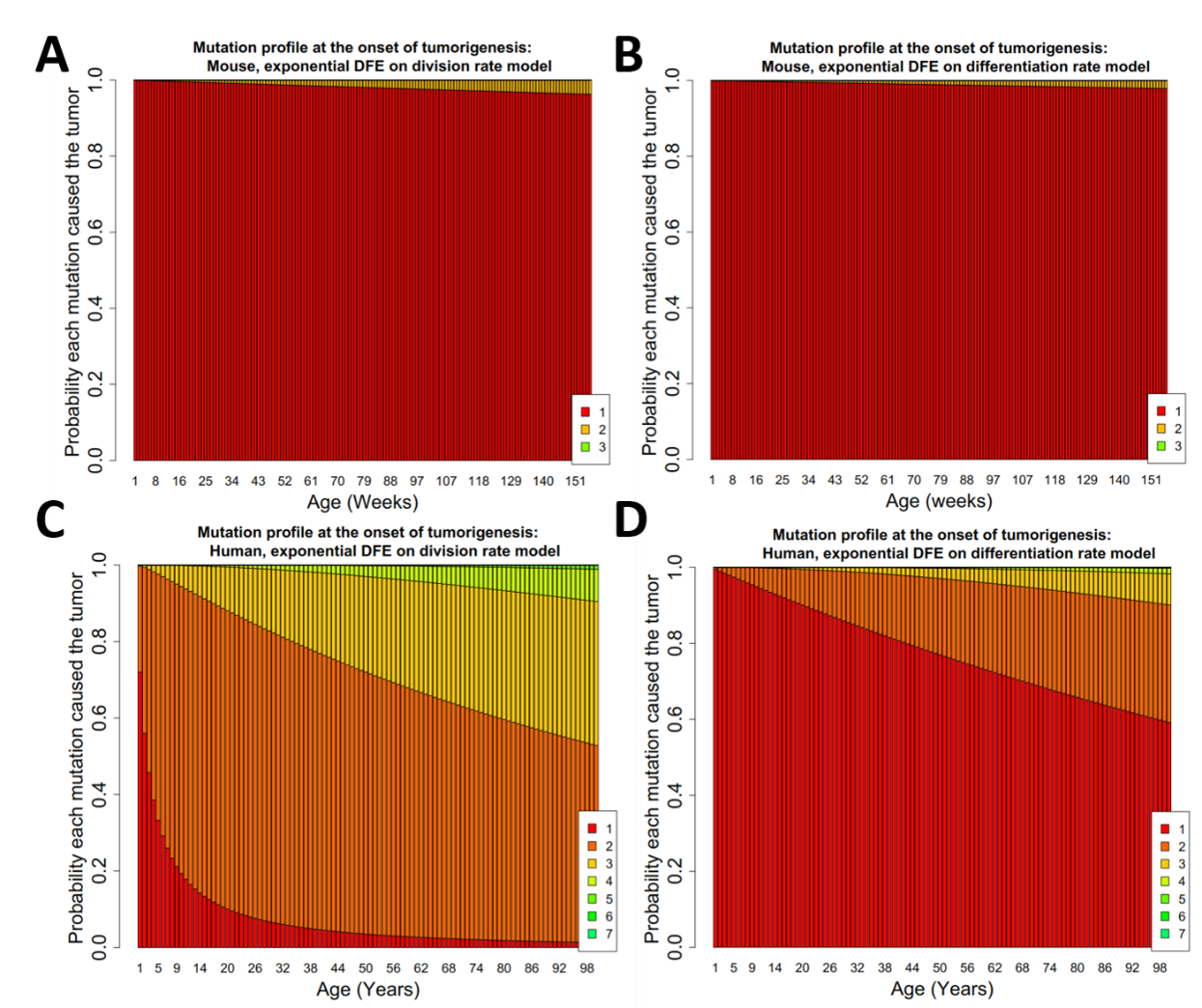

Supplement: Supplementary file 4 — Figure S4. Mutation profiles of a tumor at the onset of tumorigenesis. [file EVA-9-565-s004.png]

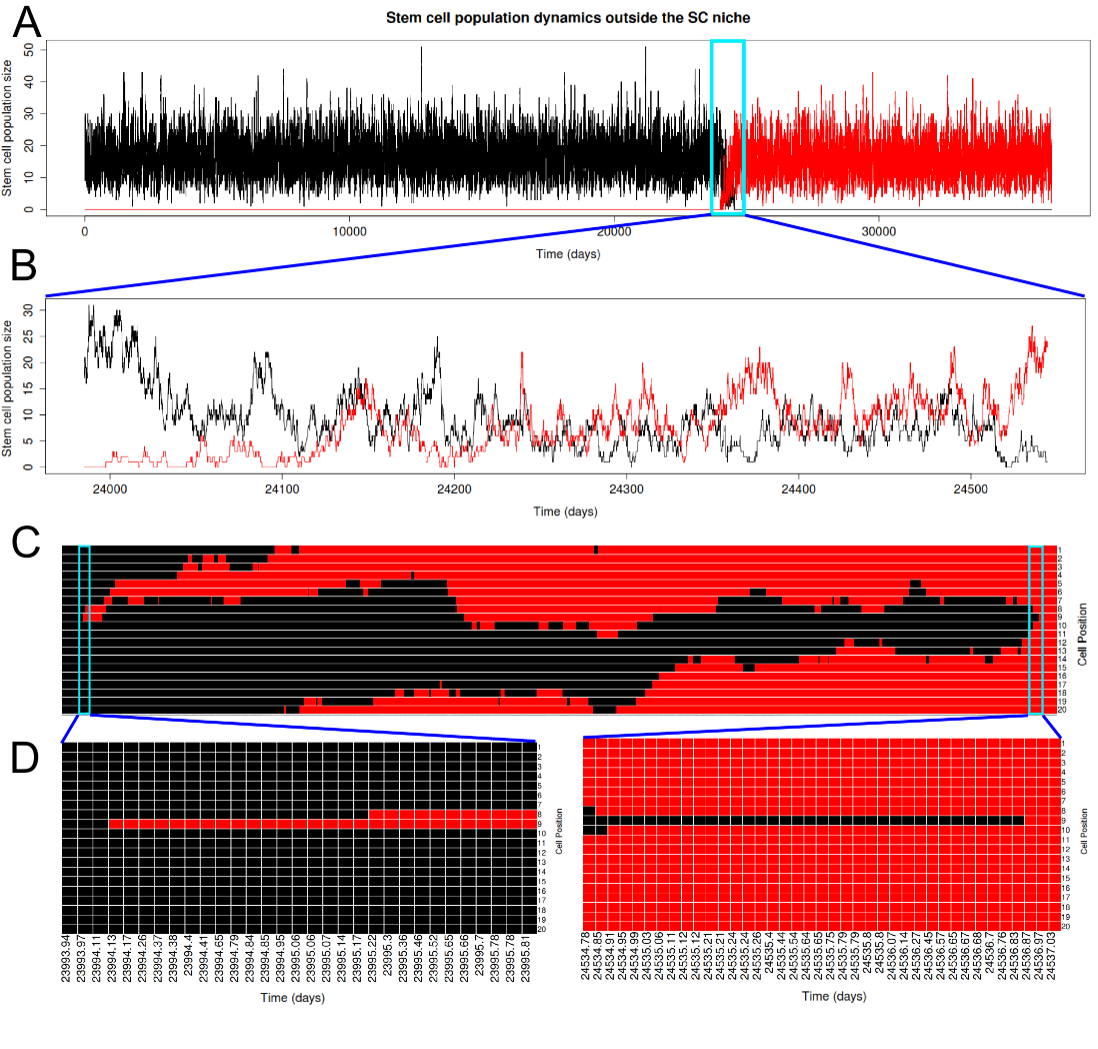

Supplement: Supplementary file 5 — Figure S5. The simulated stem cell dynamics within a human crypt for both the displaced stem cells (A,B) and the stem cell niche (C,D) showing a fixation event of a mutant lineage. [file EVA-9-565-s005.png]
